# Tolerogenic versus Inflammatory Activity of Peripheral Blood Monocytes and Dendritic Cells Subpopulations in Systemic Lupus Erythematosus

**DOI:** 10.1155/2012/934161

**Published:** 2012-08-30

**Authors:** Tiago Carvalheiro, Ana Rodrigues, Ana Lopes, Luís Inês, Isabel Velada, Andreia Ribeiro, António Martinho, José A. P. Silva, Maria L. Pais, Artur Paiva

**Affiliations:** ^1^Histocompatibility Centre of Coimbra, Edifício São Jerónimo, 4 Piso, Praceta Mota Pinto, 3001-301 Coimbra, Portugal; ^2^College of Health Technology of Coimbra, S. Martinho do Bispo, 3046-854 Coimbra, Portugal; ^3^Rheumatology Department, University Hospital of Coimbra, 3000-075 Coimbra, Portugal; ^4^Faculty of Health Sciences, University of Beira Interior, 6200-506 Covilhã, Portugal; ^5^Faculty of Medicine, University of Coimbra, 3004-504 Coimbra, Portugal

## Abstract

Abnormalities in monocytes and in peripheral blood dendritic cells (DC) subsets have been reported in systemic lupus erythematosus (SLE). We aim to clarify the tolerogenic or inflammatory role of these cells based on ICOSL or IFN-**α** and chemokine mRNA expression, respectively, after cell purification. The study included 18 SLE patients with active disease (ASLE), 25 with inactive disease (ISLE), and 30 healthy controls (HG). In purified plasmacytoid DC (pDC) was observed a lower ICOSL mRNA expression in ASLE and an increase in ISLE; similarly, a lower ICOSL mRNA expression in monocytes of ALSE patients was found. However, a higher ICOSL mRNA expression was observed in ASLE compared to HG in myeloid DCs. Interestingly, clinical parameters seem to be related with ICOSL mRNA expression. Regarding the inflammatory activity it was observed in purified monocytes and CD14^−/low^ CD16^+^ DCs an increase of CCL2, CXCL9, and CXCL10 mRNA expression in ASLE compared to HG. In myeloid DC no differences were observed regarding chemokines, and IFN-**α** mRNA expression. In pDC, a higher IFN-**α** mRNA expression was observed in ASLE. Deviations in ICOSL, chemokine, and IFN-**α** mRNA expression in peripheral blood monocytes and dendritic cells subpopulations in SLE appear to be related to disease activity.

## 1. Introduction

Systemic lupus erythematosus (SLE) is a multisystemic disease resulting from an abnormal immunological function that affects several organ systems characterized by a broad spectrum of clinical manifestations and a multitude of cellular abnormalities. The primary pathological findings in SLE patients are inflammation, vasculitis, immune complex deposition, and vasculopathy [1–3]. The exact etiology still remains unclear; however defective clearance of apoptotic material and/or aberrant apoptosis, in combination with susceptible genetic background have been suggested to be involved in SLE development and progression [4–6].

SLE patients exhibit numerous aberrations in the immune system, comprising B cells, T cells, monocytes, and dendritic cells, resulting in B and T cell activation and consequent autoantibodies production against a large variety of autoantigens [2]. 

Abnormalities in monocyte phenotype and function have been identified in several autoimmune disorders, including SLE, which could contribute to disease pathogenesis [7, 8]. Likewise, dendritic cells (DCs) subsets are also implicated in SLE pathogenesis and progression [4, 9]. Recent studies have described alterations in the number of peripheral blood (PB) DCs, namely myeloid (mDC) and CD14^−/low^CD16^+^ subsets, in their ability to produce inflammatory cytokines, activation status, and chemokine receptors expression [10, 11]. 

The immunologic self-tolerance breakdown, particularly in the control of self- and non-self-discrimination, results in the development of autoimmune diseases. Therefore, elucidate the mechanisms that regulate self-tolerance is important to understand self-directed immune responses and the mechanisms underlying autoimmune diseases [12, 13]. The notable functional plasticity of DCs, their lineage and maturational status, stimulation by pathogen-derived products, the net effect of antigen dose, and cytokine milieu determine whether an immunogenic or tolerogenic response will be developed [14].

One important mediator of DCs tolerogenic activity is ICOSL (inducible costimulator ligand), which is mainly expressed in pDC, mDCs, immature B cells, and monocytes and appears to be involved in the induction of a suppressive effect in T cells under an inflammatory environment as seen in SLE [15]. ICOS is a costimulator molecule expressed on CD4+ T cells, which was associated with secretion of interleukin 10 (IL-10) [15–17]. IL-10 is produced by T cells and induces tolerance and anergy in effector T cell [18]. ICOS is expressed at high levels in Th2 and at low levels in Th1 cells and the expression of this molecule inhibits the secretion of IL-2 [16]. The activation of ICOS/ICOSL pathway induces a differentiation of effector T cells in regulatory T cell and a sustained Th2 response [19, 20].

SLE is characterized by an inflammatory immune response mediated, in part, by cytokines and chemokines produced by antigen presenting cells (APC) and other immune cells, contributing for disease development and progression.

Multiple links of evidence support the involvement of IFN-*α* in the primary pathogenesis of SLE; high levels of serum IFN-*α* have been detected in SLE patients and have long been related with SLE pathogenesis [21]. Plasmacytoid DC (pDC) subpopulation is an important mediator of antiviral immunity through their extraordinary ability to secrete high levels of IFN-*α* in response to many DNA and RNA viruses and, in this sense, has been closely related to SLE physiopathology [22, 23].

There is a growing evidence suggesting that infiltration of T lymphocytes and other leukocytes into the sites of inflammation plays a critical role in organ involvement in SLE [24]. Chemokines have an important role in the migration and homing, necessary for the initiation of a cellular immune response in the sites of inflammation, and are able to regulate a differential recruitment of T helper (Th1 and Th2) lymphocytes [25].

Alterations in the cytokine and chemokine profile in SLE patients compared to normal controls have been described and reflect alterations in the inflammatory environment [2, 26, 27]. Chemokines like CCL2, CXCL10, CXCL9, CCL4, and CCL5 present raised levels in SLE patients serum and may be related to disease activity, contributing to the inflammatory disorder [28, 29].

In this context, we evaluated the regulatory function of peripheral blood monocytes, mDCs, CD14^−/low^CD16^+^ DCs, and pDCs subsets by the ICOSL mRNA expression and, on the other hand, we assessed the inflammatory role of these cells by the mRNA expression of IFN-*α* and the chemokines CCL2, CXCL9, CXCL10, CCL4, and CCL5.

## 2. Methods

### 2.1. Patients and Samples

Forty-three SLE patients were enrolled in the study, eighteen with active disease (ASLE) (100% female, mean age 33 ± 11 years) and twenty-five with inactive disease (ISLE) (84% female, mean age 33 ± 10 years). Patients were recruited fulfilling the 1997 American College of Rheumatology (ACR) classification criteria for SLE [30]. All patients are followed at the Lupus Clinic, Rheumatology Department of the University Hospital of Coimbra. After assessing disease activity at the time of evaluation, according to the SLE Disease Activity Index 2000 (SLEDAI 2k) [30, 31], SLE patients were divided into two groups, one with active (SLEDAI 2*k* ≥ 5; *n* = 18) and the other with inactive (SLEDAI 2*k* < 5; *n* = 25) SLE [32]. The patients medication, at time of evaluation and additional clinical and therapeutic regimen, was recorded at the time of analysis (Table 1).

The healthy control group (HG) consisted of 30 healthy individuals (90% female; mean age 30 ± 6 years). These participants were required to complete a brief questionnaire regarding previous or current medical conditions. All were free from autoimmune disease, active inflammatory condition and were not undergoing treatment with any immunomodulatory drugs.

K3-EDTA-anticoagulated peripheral blood samples were collected from each participant and FACS-sorted within 18 hours after collection.

### 2.2. Ethics

The study protocol was approved by the local ethics committee. All participants gave and signed informed consent and the principles of Helsinki Declaration were respected.

### 2.3. Cell Sorting of Monocytes, CD14^−/low^CD16^+^DC, mDCs, and pDCs

For the cell sorting of monocytes, CD14^−/low^CD16^+^ DC, mDCs, and pDCs, 3 mL of each K3-EDTA PB sample were added to 10 mL of NH_4_Cl solution (Sigma, St. Louis, MO, USA) in order to lyse red blood cells. After 20 minutes of incubation, samples were centrifuged (5 minutes, at 540 ×g) and the cell pellet was stained with the following monoclonal antibodies (mAb): anti-CD16 fluorescein isothiocyanate (FITC) (Sanquin–Pelicluster, Amsterdam, The Netherlands), anti-CD33 phycoerythrin (PE), anti-CD45 peridinin chlorophyll protein (PerCP) (BDB, San Jose, CA, USA), anti-HLA-DR phycoerythrin cyanine 7 tandem (PECy7) (BDB), and anti-CD123 allophycocyanin (APC) (Macs Miltenyi Biotec, Bergisch Gladbach, Germany). Once incubated for 20 minutes at room temperature in the darkness, the cells were washed and resuspended in phosphate-buffered saline (PBS) (Gibco BRL-life Technologies, Vienna, Austria).

**Figure 1:**
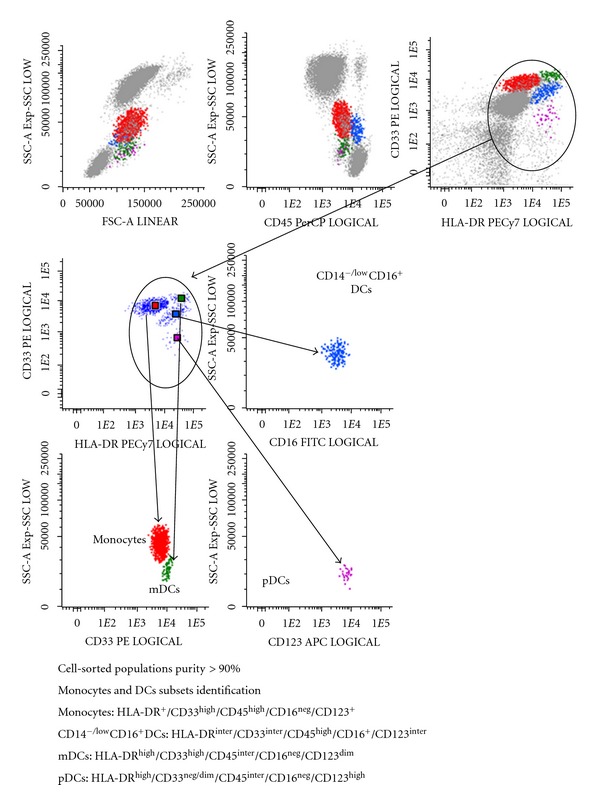
Flow cytometry gate strategy to obtain purified monocytes and peripheral blood dendritic cells by cell sorting.

Cell sorting and purification were performed in FACSAria II cell sorter (BDB) using the FACSDiva software (BDB). Monocytes were identified and sorted by HLA-DR^+^/CD33^high^/CD45^high^ phenotype, and the three DCs subpopulations, characterized by intermediate forward (FSC) and side scatter (SSC) between those of lymphocytes and monocytes, were purified according to the following immunophenotype features: myeloidDCs (mDCs) present HLA-DR^high^/CD33^high^/CD16^neg^/CD123^dim⁡^ immunophenotype, CD14^−/low^CD16^+^ DC subset are HLA-DR^inter^/CD33^inter^/CD123^inter^, and plasmacytoid DCs (pDC) are HLA-DR^high^/CD123^high^CD33^neg/dim⁡^/CD16^neg^ (Figure 1) [33, 34]. The number of cells obtained of each cell population after FACSAria cell sorting is described in Table 2.

After cell sorting, the purity of the isolated cell populations was evaluated in the FACSCanto II flow cytometer (BDB) using the FACSDiva software (BDB) and acquiring a representative number of sorted cells, and it was consistently greater than 90%.

### 2.4. Gene Expression Analysis after Sorting of Monocytes, Dendritic Cells Subsets

Sorted cell populations were centrifuged for 5 minutes at 300 g and the pellet was resuspended in 350 *μ*L of RLT Lysis Buffer (Qiagen, Hilden, Germany) and the total RNA extraction was performed with the RNeasy Micro kit (Qiagen) according to the supplier's instructions. Total RNA was eluted in a 14 *μ*L volume of RNase-free water. In order to quantify the amount of total RNA extracted and verify RNA integrity, samples were analyzed using a 6000 Nano Chip kit, in an Agilent 2100 bioanalyzer (Agilent Technologies, Waldbronn, Germany) and 2100 expert software, according to the manufacturer's instructions. RNA was reverse transcribed with SuperScript III First-Strand Synthesis SuperMix for qRT-PCR (Invitrogen, Carlsbad, CA, USA), according to the manufacturer's instructions. Relative quantification of gene expression by real-time PCR was performed in the LightCycler 480 II (Roche Diagnostics, Rotkreuz, Switzerland). Real-time PCR reactions were carried out using 1X QuantiTect SYBR Green PCR Master Mix (Qiagen), 1X QuantiTect Primer Assay (IFNA1 QT00201964, ICOSLG QT00004669, CCL2 QT00212730, CCL4 QT01008070, CCL5 QT00090083, CXCL9 QT00013461, and CXCL10 QT01003065) (Qiagen), and 20 ng of cDNA sample, in a total volume of 10 *μ*L. The reactions were performed using the following thermal profile: 15 min at 95°C, 50 cycles of 15 sec at 94°C, 30 sec at 55°C, and 30 sec at 72°C. Melting point analysis was done to ensure amplification of the specific product. Real-time PCR results were analyzed with the LightCycler software (Roche Diagnostics). GeNorm Reference Gene Selection kit (Primer Design Ltd., Southampton, UK) in conjunction with the geNorm software (Primer Design Ltd.) were used to select the reference genes to normalize data. The reference genes used for gene expression analysis in monocytes were ATP synthase (ATP5B) and the beta-2-microglobulin (B2 M); in mDC and CD14^−/low^CD16^+^ DC were the B2 M and ubiquitin-c (UBC); in pDC were the B2 M and ATP5B. The normalized gene of interest expression levels were calculated by using the delta-Ct method [35].

### 2.5. Statistical Analyses

Statistical evaluation of data was analyzed using the nonparametric Mann-Whitney *U* test between the studied groups. All statistical analyses were performed using IBM SPSS statistics 20 software (Armonk, NY, USA) and differences were considered as statistically significant when the *P*  value was less than 0.05.

## 3. Results

### 3.1. Frequency of Peripheral Blood Monocytes, CD14^−/low^CD16^+^ DCs, mDCs, and pDCs in SLE Patients and Healthy Control Group

As shown in Table 3, frequency of peripheral blood mDCs and pDCs was lower in ASLE group than in control group, particularly pDCs. A lower pDC frequency was also observed in ISLE group compared to HG. In contrast, no significant differences were found in the frequency of circulating monocytes and CD14^−/low^CD16^+^ DCs. We also verified a lower absolute number of monocytes in ASLE compared to HG as well as a lower number of peripheral blood pDCs in SLE patients, especially in ALSE group. 

Since the number of dendritic cells obtained after cell sorting was significantly lower than those of monocytes, we only evaluated the mRNA expression of IFN-*α*, ICOSL, CXCL9, and CXCL10 on mDCs and CD14^−/low^CD16^+^ dendritic cells and of IFN-*α* and ICOSL on pDCs (Table 2).

### 3.2. Tolerogenic Role of Monocytes, CD14^−/low^CD16^+^ DCs, mDCs, and pDCs Based on ICOSL mRNA Expression

**Figure 2:**
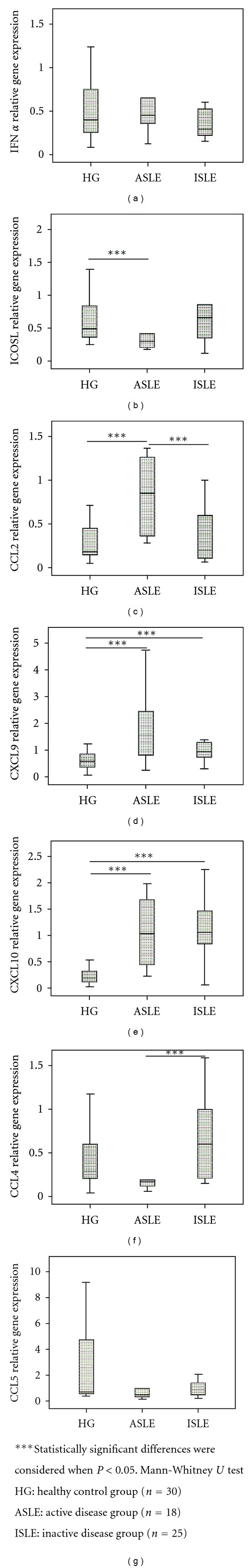
IFN-*α*, ICOSL, CCL2, CXCL9, CXCL10, CCL4, and CCL5 relative gene expression in cell-sorted monocytes in the three studied groups (HG, ASLE, and ISLE).

Concerning the tolerogenic function of monocytes and DCs subsets, a lower mRNA expression of ICOSL was observed in ASLE compared to HG in monocytes (Figure 2(b)) and, on the other hand, an increased ICOSL mRNA expression in mDCs from both SLE groups compared to HG, was found (Figure 4(a)). 

**Figure 3:**
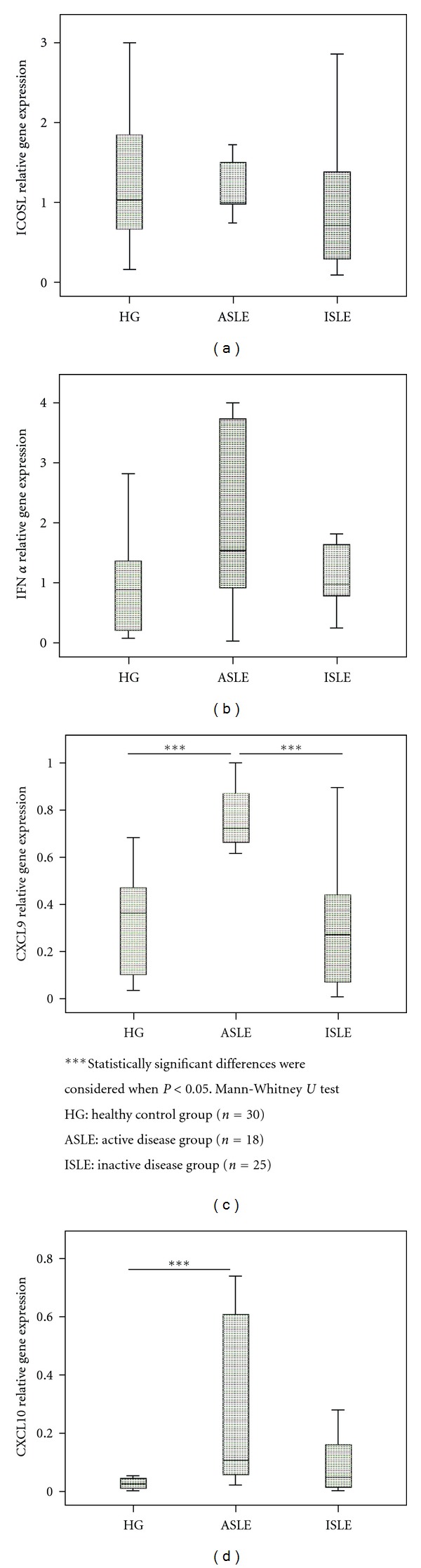
IFN-*α*, ICOSL, CXCL9, and CXCL10 relative gene expression in cell-sorted CD14^−/low^CD16^+^ DCs subset in the three studied groups (HG, ASLE, and ISLE).

No significant differences were observed in CD14^−/low^CD16^+^DC subset between the studied groups (Figure 3(a)).

Moreover, in pDC subpopulation, a lower ICOSL mRNA expression in ASLE and higher in ISLE compared to HG was observed (Figure 5(b)).

### 3.3. Inflammatory Role of Monocytes, CD14^−/low^CD16^+^ DCs, mDCs, and pDCs Based on Chemokines and IFN-*α* mRNA Expression

In purified monocytes was observed a significant increase of CXCL9 and CXCL10 mRNA expression in both SLE groups compared to HG (Figures 2(d) to 2(e)). Similarly a higher mRNA CCL2 expression was observed in ASLE compared to HG and ISLE (Figure 2(c)). Moreover CCL4 mRNA expression was higher in ISLE, reaching statistical significance when compared with ASLE group (Figure 2(f)). Regarding IFN-*α* and CCL5 mRNA expression, no differences were found between the studied groups (Figures 2(a) and 2 (g)).

In CD14^−/low^CD16^+^ DC subset a higher CXCL10 and CXCL9 mRNA expression in ASLE was noted, when compared with HG, and in the latter chemokine, when compared with ISLE (Figures 3(c) to 3(d)). The evaluation of the IFN-*α* mRNA expression did not present significant differences between the studied groups (Figure 3(b)).

**Figure 4:**
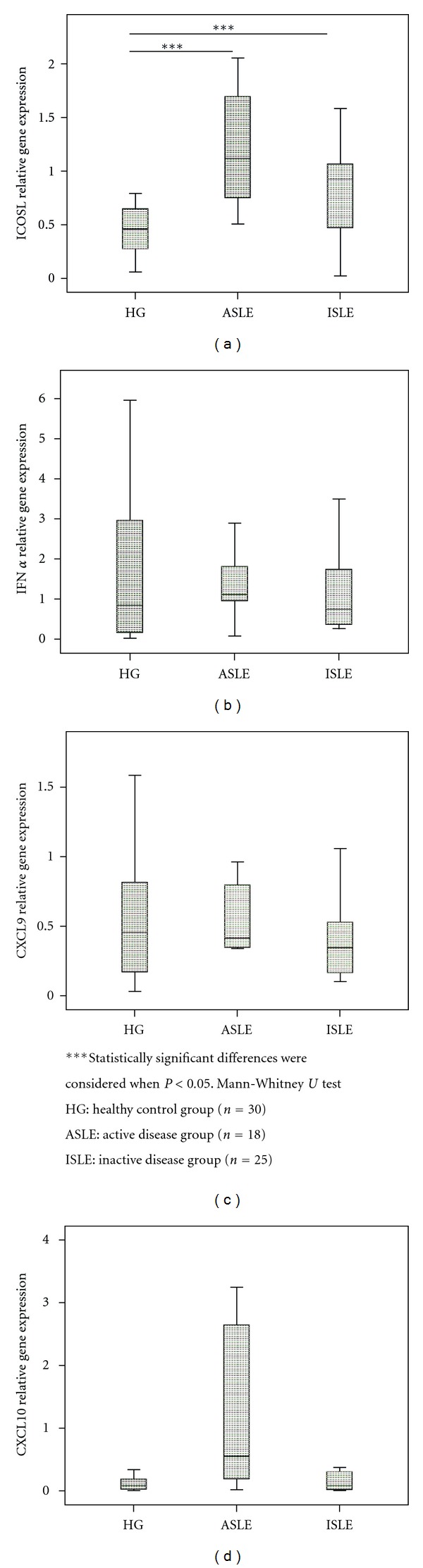
ICOSL, IFN-*α*, CXCL9, and CXCL10 relative gene expression in cell-sorted mDCs subset in the three studied groups (HG, ASLE, and ISLE).

Regarding the mDCs subpopulation, we did not found statistical significant differences for IFN-*α*, CXCL9, and CXCL10 mRNA expression between the studied groups (Figures 4(b) to 4(d)).

**Figure 5:**
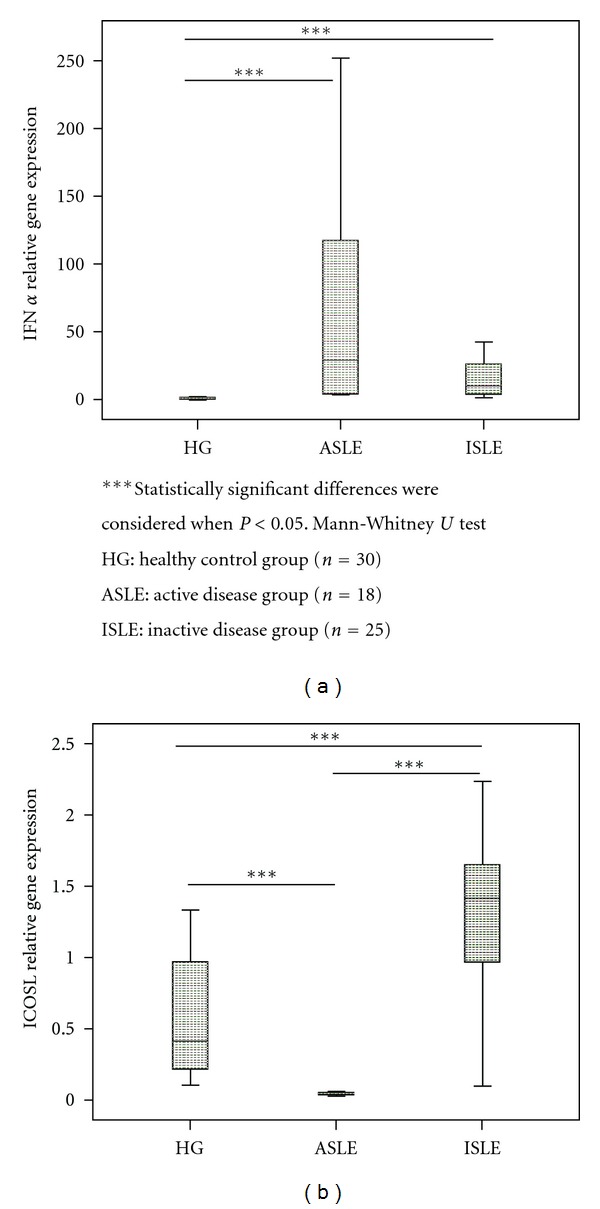
IFN-*α* and ICOSL relative gene expression in cell-sorted pDCs subset in the three studied groups (HG, ASLE, and ISLE).

IFN-*α* mRNA expression evaluated on pDC subset revealed a significant increase in both SLE groups when compared with HG, particularly in ALSE (Figure 5(a)).

### 3.4. ICOSL mRNA Expression and Clinical Parameters

**Figure 6:**
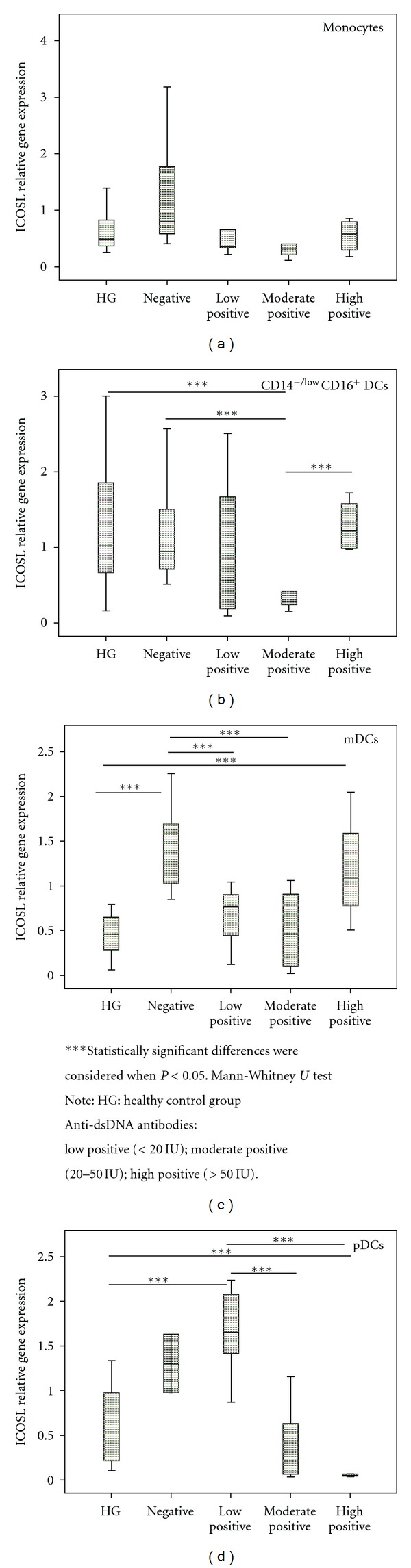
ICOSL relative gene expression in cell-sorted monocytes and DCs subsets, according to the amount of anti-dsDNA antibodies: negative; low, moderate, and high positive.

When we grouped SLE patients based on the amount of anti-dsDNA antibodies in negative, low (<20 IU), moderate (20–50 IU), and high positive (>50 IU), we found, in pDC, an increase on ICOSL mRNA expression in the groups without anti-dsDNA antibodies and lower positive, when compared with moderate and high positive groups. Inline with this observation, we also detected a significant increase of ICOSL expression in mDC on negative group and in a lower extension in high positive group, when compared with lower and moderate positive groups. Moreover, in CD14^−/low^CD16^+^DC, we found a decrease on ICOSL expression on moderate-positive group when compared with high-positive and negative groups (Figure 6).

**Figure 7:**
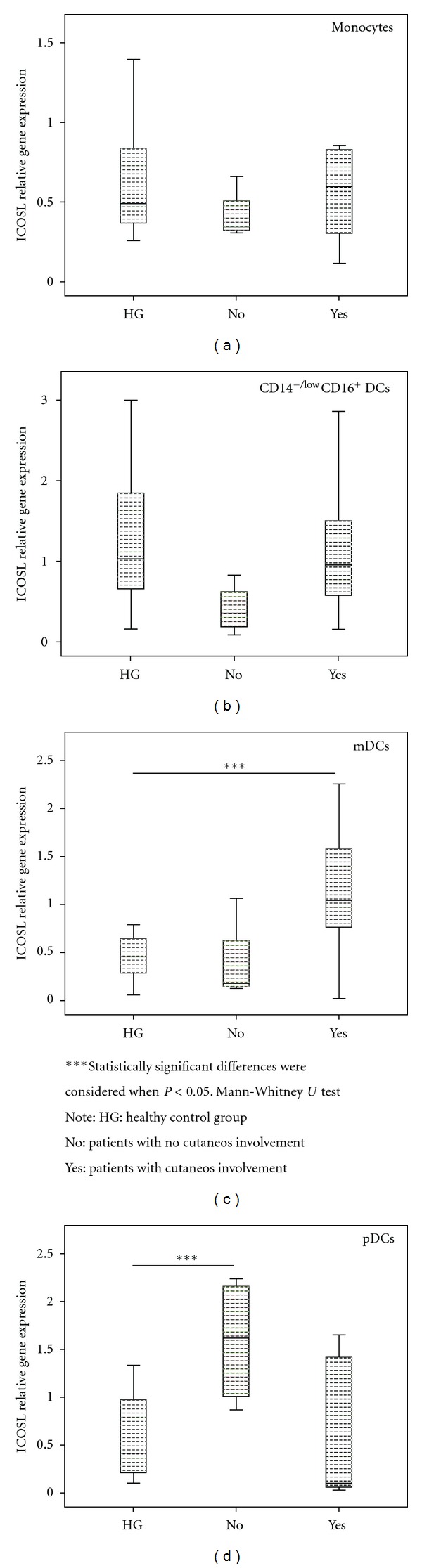
ICOSL relative gene expression in cell-sorted monocytes and DCs subsets, according to the cutaneos involvement of SLE patients.

Concerning cutaneous involvement, we found, in SLE patients without this clinical feature, an increase on ICOSL mRNA expression in pDC. Also, an increase of its expression was observed in mDC in patients with this clinical parameter (Figure 7).

No more statistical significant differences were found relating other clinical parameters and/or other studied molecules.

## 4. Discussion

Monocytes and DCs are involved in the host defense and regulation of inflammation, playing a critical role in both adaptive and innate immune responses and in tolerance development. SLE is a variable autoimmune inflammatory condition, associated to tissue destruction wherein several abnormalities and disturbances have been attributed to these cells in SLE [8, 26, 36]. 

The tolerogenic function mainly attributed to pDC is, in part, mediated by the expression of ICOSL which has the ability to generate anergy in T cells and induce differentiation of *naive* T cells into regulatory T cells [32, 37, 38].

The lower levels of ICOSL mRNA expression observed in pDC from ASLE patients could be related to the higher inflammatory peripheral environment, due to increased levels of proinflammatory cytokines and the presence of circulating immune complexes, which is inline with the higher levels of IFN-*α* mRNA found in these cells. The opposite was observed in ISLE, namely, higher mRNA expression of ICOSL and lower of IFN-*α*. This pattern of ICOSL expression in pDC was also observed in SLE patients without anti-dsDNA antibodies or with lower levels, as well as in the group of patients without skin involvement.

In fact, the lower mRNA expression of ICOSL and the mechanisms involved in ICOS/ICOSL pathway are related to loss of tolerance to self-antigens that occur in SLE, especially in patients in active phase [32, 37, 38]. It is described that the absence of interaction of ICOS with its ligand overrides the induction of anergy in T cells, considered the first step in the differentiation of T helper cells into T suppressor cells [15]. The reduction of ICOSL expression may also be explained, at least in part, by a negative feedback mechanism by which high levels of ICOS lead to the decrease of ICOSL expression. Since it was reported that active SLE patients have an increased expression of ICOS on CD4+ and CD8+ T cells, thus, apparently, exists a negative correlation between these two molecules [16, 39]. Results of Yang et al. showed a decreased expression of ICOS on CD4+ and CD8+ T cells from ISLE patients when compared with ASLE, resulting in a possible increase of ICOSL in these patients [16].

As observed in pDC, ICOSL mRNA expression in monocytes is reduced when compared to the HG, probably due to the same mechanisms observed in pDC. On contrary, high mRNA expression of this molecule was observed in mDC from ASLE and, in a lower extent, for ISLE patients when compared with control group, which could mean that this subpopulation of dendritic cells is less sensitive to the peripheral inflammatory environment, probably due to the fact that the majority of peripheral blood mDCs are recent immigrates from bone marrow with an immature phenotype, which could be particularly true in SLE patients, where an increase migration of these cells to peripheral tissues could induce an increase in the hematopoiesis of this cell lineage [40, 41]. In line with this explanation is the fact that no statistical significant differences were observed in this cells for IFN-*α* and chemokines mRNA expression among the studied groups. Furthermore the more immature status of mDC could be also the explanation for the higher mRNA expression of ICOSL found in patients with skin involvement, to where occurs an increased mDC migration.

Previous data have reported elevated levels of IFN-*α* in the SLE patient's serum [42, 43], which is in agreement with the higher mRNA expression of this cytokine in pDC from SLE patients, particularly in those with active disease. Dall'Era et al. and Kirou et al. related the serological levels of IFN-*α* with SLE clinical manifestations and disease activity [42, 44].

IFN-*α* is a pleiotropic cytokine, important in the immune regulation, that is produced by multiple cell types in response to viral infection. pDCs have a special role in the IFN-*α* production and are the most important sources of serum interferon [45]. IFN-*α* can affect multiple cell types involved in SLE and has the potential to influence the development, progression, and pathogenesis of SLE as it can control the function and activation states of most important immune cell subsets and function as a bridge between innate and adaptive immunity [46].

Some studies have demonstrated that the frequency of circulating pDCs is markedly reduced in SLE patients [47, 48]. However, functional studies revealed that pDCs, upon stimulation, have a normal IFN-*α* producing capacity, which means that aberrant pDC activation may be an important step in autoimmune diseases like SLE. In fact, an important finding was that the immune complexes present in SLE patients serum contain nucleic acids that are internalized via the Fc*γ*RIIa, reach the endosome, and stimulate TLR7 and/or TLR9, leading to subsequent activation of transcription factors and IFN-*α* production [49, 50].

Several studies have revealed the important role of chemokines and IFN-*α* in SLE activity. Many have reported high levels of those in the serum as well as of mRNA chemokine expression in peripheral blood leukocytes of these patients, particularly in active disease [29, 51, 52]. DCs subtypes have individual functions and appear to influence multiple processes that may activate or regulate autoreactive B cells. Part of their influence is dictated by their receptors and cytokines profiles and also by their location [9]. In the present study the use of purified peripheral blood monocytes and DCs subpopulations emphasizes the role of these cells in SLE pathophysiology, based on their chemokine expression.

The altered chemokines mRNA expression observed on monocytes in SLE patients, namely, in ASLE, is in accordance with the abnormalities already observed in these patients [8, 53]. The high levels of CCL2, CXCL9, and CCL4 mRNA expression observed on monocytes from SLE patients are consistent with other reports that have found increased levels in serum from these patients [52, 54]. These findings may be associated to the IFN-*α* pathway, since higher levels of IFN-*α* have been associated with increased levels of chemokines in SLE patients, suggesting an upregulation of this chemokine production according to Bauer et al. studies [28, 54]; likewise Quiong Fu has suggested the importance of type I IFN system in modulating chemokine expression, linking these two networks in the SLE pathogenesis [55].

Moreover, the inflammatory environment of SLE may lead to chemokine imbalance, including monocyte mobilization. CCL2 is involved in monocyte recruitment into focus of active inflammation and may act as a potent factor in the polarization of Th0 cells toward a Th2 phenotype [56]. In turn, there is increasing evidence that CXCL10 levels are elevated in serum and in tissues of SLE patients, contributing to a large variety of SLE manifestations [57]. Furthermore, according to Kong et al. data, CXCL10 levels correlate positively with SLE disease activity and may represent a fair marker for monitoring disease activity [58]. As reported by Karonitsch et al., CXCL10 and CXCL9 mRNA expressions in monocytes were increased in SLE patients, associated with increased responsiveness of monocytes to IFN-*γ*, confirmed by mRNA levels of IFN-inducible STAT-1–dependent CXCL10 and CXCL9 genes [59]. 

Like monocytes, CD14^−/low^CD16^+^ DC subpopulation presented higher levels of CXCL9 and CXCL10 mRNA expression in ASLE group. This data point to a common role of these cells in SLE pathophysiology, as we previously reported [10].

Apparently less sensitive to microinflammatory changes than monocytes, CD14^−/low^CD16^+^ DC express Fc*γ*RII CD16^+^ [60], which allow these cells to respond to peripheral activators motifs like circulating immune complexes. Moreover, these cells are tissue derivated, reentering in the peripheral circulation, as previously reported [61, 62], reflecting in the periphery the tissue injure.

As we previously described, no significantly differences on CXCL9 and CXCL10 mRNA expression in mDC were observed in SLE patients, when compared with the control group. In agreement with our data, Gerl et al. reported no differences in the expression of CCR7, CCR1, and CCR5 chemokine receptors in mDC from SLE patients [11]. 

In conclusion our data clearly demonstrates a different role for monocytes and DCs subsets in SLE pathophysiology.

 In active disease, peripheral blood monocytes and CD14^−/low^CD16^+^ DCs exhibit an upregulation of chemokine expression, probably due to a higher activation status in the periphery, contributing to the recruitment of neutrophils, monocytes/macrophages, and T and NK cells to peripheral tissues.

In turn, pDCs upregulate IFN-*α* and downregulate ICOSL mRNA expression in ASLE, exhibiting a pro-inflammatory profile and, conversely, in ISLE they seem to display a more tolerogenic activity.

## Figures and Tables

**Table 1:** Clinical findings in 43 patients with systemic lupus erythematosus (SLE).

	ASLE	ISLE
	(*n* = 18)	(*n* = 25)
Mean SLEDAI scores	9.7 ± 3.2	1.6 ± 0.9
Mean time since diagnosis	7.6 ± 7.4	9.0 ± 6.0
Lupus nephritis	44.4%	61.3%
Neurolupus	0%	19.4%
Lupus arthritis	66.7%	58.1%
Haematological involvement	100%	87.1%
Lupus cutaneous involvement	77.8%	74.2%
Severe Lupus*	44.4%	71%
Anti-dsDNA antibodies**		
Low positive	11.1%	32.3%
Moderately positive	22.2%	22.6%
High positive	55.6%	6.5%
Treatment		
Hydroxychloroquine	94.4%	87.1%
Immunossupressants***	66.7%	32.3%
Steroids****	83.4%	12.9%
Low dose	46.6%	100%
Moderate dose	33.3%	0%
High dose	20.1%	0%

ASLE: Active disease group.

ISLE: Inactive disease group.

*Lupus severity in accordance with cumulative major organ involvement.

**Anti-dsDNA antibodies: low positive (<20 IU); moderately positive (20–50 IU); high positive (>50 IU).

***Azathioprine, mycophenolate mo1etil, cyclosporine, tacrolimus, methotrexate, cyclophosphamide, or rituximab.

****Low dose, upto 10 mg/day; moderate dose, 10–30 mg/day; high dose, more than 30 mg/ day; *n* = sample investigated.

**Table 2:** Number of sorted monocytes and peripheral blood dendritic cells in the three studied groups (HG, ASLE, and ISLE).

	HG	ASLE	ISLE
	(*n* = 30)	(*n* = 18)	(*n* = 25)
Number of sorted cells			
Monocytes	143701 ± 110950	91029 ± 83915	115407 ± 10558
CD14^−/low^CD16^+^ DCs	15393 ± 18486	9667 ± 11976	7251 ± 3903
mDCs	8709 ± 7107	4365 ± 3228	3771 ± 3076
pDCs	5281 ± 3894	1363 ± 1291	3416 ± 2655

HG: Healthy control group.

ASLE: Active disease group.

ISLE: Inactive disease group.

**Table 3:** Frequency and absolute value of monocytes and peripheral blood dendritic cells in the three studied groups (HG, ASLE, and ISLE).

	HG	ASLE	ISLE
	(*n* = 30)	(*n* = 18)	(*n* = 25)
Frequency (%)			
Monocytes	3.9 ± 0.97	3.02 ± 1.61	3.56 ± 1.32
CD14^−/low^CD16^+^ DCs	0.54 ± 0.29	0.45 ± 0.30	0.55 ± 0.33
mDCs	0.29 ± 0.18*	0.21 ± 0.15**	0.29 ± 0.32
pDCs	0.10 ± 0.07*	0.02 ± 0.03	0.07 ± 0.07***
Absolute Value (cells/*μ*L)			
Monocytes	284,6 ± 84,2*	193,3 ± 97,5	228,4 ± 87,1
CD14^−/low^CD16^+^ DCs	39,2 ± 23,2	28,1 ± 20	34,1 ± 19,1
mDCs	21,4 ± 15,1	13,9 ± 11,1	18,2 ± 14
pDCs	7.08 ± 5.16*	1.24 ± 1.28**	3.82 ± 3.51***

Note: results are expressed as mean ± standard deviation.

Statistically significant differences were considered when *P* < 0.05 (Mann-Whitney *U* test): *HG versus ASLE; **ASLE versus ISLE., ***HG versus ISLE.

HG: healthy control group.

ASLE: active disease group.

ISLE: inactive disease group.
